# Bringing history back to culture: on the missing diachronic component in the research on culture and cognition

**DOI:** 10.3389/fpsyg.2015.00716

**Published:** 2015-05-27

**Authors:** Rumen I. Iliev, bethany l. ojalehto

**Affiliations:** ^1^Ford School of Public Policy, University of MichiganAnn Arbor, MI, USA; ^2^Department of Psychology, Northwestern UniversityEvanston, IL, USA

**Keywords:** culture, cognition, causality, diachronic analysis, automated text analysis

## Introduction

A growing body of evidence shows that cognitive processes in general, and causal cognition in particular, are variable across cultures (Choi et al., 1999; Norenzayan and Heine, 2005; Henrich et al., 2010). The majority of these findings are based on cross-cultural comparisons contrasting well-defined groups, with little explicit consideration of temporal change within those groups. While this strategy has undoubtedly proven successful, an important limitation is that it can implicitly lead to a view of cultures as stable entities and associated cognitive processes as essentialized.

A prosaic illustration serves to introduce this idea. Suppose we hypothesize that smoking cigarettes and culture are closely related. We measure the number of cigarettes per capita and find that Chinese smoke more than Americans (Ng et al., 2014). If we collect time-series data, however, we might notice that had our measurements been taken in 2000, we would have found no cultural difference. Further, if our time-series had gone even further back to measurements taken in the 1980s, we would have found just the opposite pattern, such that Americans smoked more than the Chinese. Clearly, findings for cultural differences are of limited utility when they do not account for within-culture historical trends. Furthermore, theoretical explanations for cultural difference risk reifying an incomplete perspective if they take such results as indicative of some atemporal notion of “culture” itself.

In this paper we argue for the need to develop methods of within-culture diachronic analysis as a necessary step for understanding the complex links between culture and cognition. We specifically focus on the link between culture and causal cognition, yet the argument is applicable to the field as a whole.

## A brief history of cultural causal cognition

Seminal work in culture and cognition focused on comparisons between Easterners (mainly Chinese, Japanese and South Koreans) and Westerners (mainly North Americans and Western Europeans).^1^ Using this East-West framework as the comparative lens, researchers found that Easterners use a holistic mode of thinking while Westerners use an analytic mode. Holistic thinking relies less on formal rules, attends to the whole field, and is more open to dialectic contradiction than analytic thinking (Nisbett et al., 2001; Nisbett and Miyamoto, 2005). In terms of causality, holistic thinkers are more likely to attend to external forces and group patterns over internal dispositions (Morris and Peng, 1994); less likely to use decontextualized information when making inferences (Norenzayan et al., 2002); consider a larger number of possible causes (Choi et al., 2003); are more sensitive to covariance and perceive stronger associations between events (Ji et al., 2000); are more likely to expect change from prior trends (Ji et al., 2001); and tend to see causes as extending further in a system (Maddux and Yuki, 2006).

In a move that proved remarkably generative, researchers theorized that contemporary East-West cognitive differences reflected epistemological orientations that could be traced back to Ancient Chinese and Greeks. Much like contemporary Eastern individuals, ancient Chinese philosophers were interested in continuity, changes, transformations, and dependence between objects, while Greek philosophers were interested in universal truths, formal rules, and discrete objects and entities. Further, ancient Chinese were interested in technological advances driven by pragmatic goals, while the Greeks valued intellectual endeavor for its own sake and were less concerned with concrete applications of knowledge (Norenzayan and Nisbett, 2000; Nisbett et al., 2001; Nisbett, 2003). Such parallels between historical philosophies and contemporary cognitive patterns subsequently informed a major line of comparative research.

This historical explanation assumes considerable psychological continuity within cultures across time. Yet accumulating evidence has begun to challenge the strong claim that contemporary cultural differences are rooted in ancient East-West philosophies (Varnum et al., 2010). First, when a group changes its environment, it may start to resemble other groups in similar environments rather than their original ancestors. Kitayama et al. (2006), for example, showed that settlers in Hokkaido in northern Japan were more individualistic and made more dispositional causal inferences than people from mainland Japan. The authors suggested that the Hokkaido's hostile environment fostered a “frontier spirit” similar to the spirit of North American settlers from 19th century. Second, within-culture differences in analytic and holistic processing closely mimic the original East-West distinction. Within the U.S., Na et al. (2010) found that working class Americans were more holistic and less analytic than middle class Americans. At minimum, this suggests that variation in cognitive orientations is not unique to East-West cultural differences. Third, cultures that share little with ancient Chinese in terms of epistemological frameworks are still found to share many features of a holistic cognitive style. Russians, for example, are historically closer to the Greek intellectual tradition but are holistic in terms of categorization, causal attribution, and reasoning about change (Grossmann, 2009, cited in Varnum et al., 2010). At minimum, these findings show that variation in holistic vs. analytic cognitive orientations are not unique to historical East-West cultural differences.

If cognitive orientations also vary within cultures that share a single Ancient tradition, then this points to shifting cognitive patterns across time. On this perspective, a fuller account of the links between culture and cognition can be realized by including within-culture comparisons aimed at mapping historical trends.

## Diachronic approaches to cultural cognition

Adding an explicit diachronic dimension to current work on culture and cognition raises many methodological and theoretical questions. Methodologically, the main challenge facing researchers interested in within-culture historical trends is the lack of longitudinal data. Part of the problem is that cognitive psychologists, especially those working on causality, often pursue very specific theoretical questions and rarely use standardized measures or common procedures. Other fields are in a more advantageous position, having collected continuous time-series data long enough to detect trends. One widely debated example comes from research on general intelligence (Teasdale and Owen, 2005; Flynn, 2007), where scientists have detected a stable increase during the last century (Flynn, 1984, 1987). Similarly, social psychologists working in the U.S. have detected increases in self-esteem (Twenge and Campbell, 2001) and decreases in conformity (Perrin and Spencer, 1981; Bond and Smith, 1996), need for social approval (Twenge and Im, 2007) and trust (Putnam, 1995; Robinson and Jackson, 2001) over the last half century. Over the same relatively short period, clinical psychologists have observed reliable increases in depression and other psychopathologies (Twenge et al., 2010) and decreased empathy and perspective taking (Konrath et al., 2011). In short, data from standardized intelligence tests, personality measures, and laboratory experiments suggest that Americans are changing on some major psychological variables.

Unfortunately, comparable data is not readily available to investigate similar shifts in cognitive dimensions. Longitudinal designs are rare among cognitive psychologists, but there are some documented cases of within-culture cognitive change. For example, in 1969/70 Patricia Greenfield brought a complex weaving task to Maya children and young adults in Mexico. Twenty years later she revisited the community and ran the same task with the same age groups (Greenfield et al., 2003). As compared to the earlier sample, the new participants demonstrated more abstract thinking and greater propensity for novelty.

Another example comes from work on cognitive models of the environment and folkbiological reasoning with several cultural groups in the Peten rainforest in Guatemala (Medin and Atran, 2004). In the 1990s, the indigenous Itza' Maya dwellers demonstrated elaborate knowledge of plant-animal interactions within a belief system oriented toward ecological centrality (Atran et al., 1999). In a second round of data collection about a decade later, researchers found that the younger generation of Itza' Maya had less folkbiological expertise than their parents, and that Itza' Maya values had shifted away from ecological centrality and toward monetary incentives (Le Guen et al., 2013).

Such examples of research documenting cognitive changes within a culture are rare, because there is no systematic longitudinal data for the vast majority of cognitive tasks (Greenfield et al., 2003, p. 456). Moreover, both aforementioned studies covered relatively short time periods where cognitive changes were attributed to abrupt socio-economic shifts within local communities. Exploring other, more gradual, forms of cultural change may require data from longer time periods to detect reliable cohort differences. Unfortunately, identical cognitive tasks are rarely used across studies, and when they are, the two measurements are rarely distant enough in time for longitudinal analysis.

Given this dearth of data, it becomes important to consider alternative approaches. Are there methods that can yield proxy measures of psychological variables when direct measurements are not available? The answer to such a question is limited only by the creativity of the researchers. One approach is to analyze ethnographic reports from different time periods and to draw inferences about a particular cultural group based on the descriptions. In one such analysis, Widlok (2014) scrutinized ethnographic accounts to provide evidence for cultural systems of causal cognition observed across many decades. Another promising method comes from analysis of cultural products, such as magazines, advertisements, websites and news coverage (see Morling and Lamoreaux, 2008). For more distant periods where ethnographic description is unavailable, a researcher might analyze extant artifacts such as tools, for example, and offer some hypotheses about how previous humans represented causality and reasoned about agency, goals and cause-effect relation (Haidle, 2014; see also Alberti and Bray, 2009).

More recently, automatic text analysis has extended classical anthropological and archeological methods to infer psychological variables. Various methods of automated text analysis are now in use by psychologists (Iliev et al., 2015). In dictionary-based methods, which are the most straightforward to apply, researchers assemble a set of words related to a particular variable of interest. For example, positive affect in a text might be measured by constructing a dictionary of terms such as “happiness,” “joy,” “cheerful,” “optimism,” etc. The relative frequency of target words across texts can then be used to test hypotheses about positive affect associated with different texts across time, for instance. While researchers can assemble their own dictionaries, many social scientists have begun using a dedicated software application called LIWC (Pennebaker et al., 2001), which consists of multiple categories oriented around general topics such as social processes, affect, cognition, perception, and grammatical features of language.

Automated text analysis can be particularly useful for detecting historical trends in large corpora. One compelling demonstration comes from a study by Wolff et al. (1999), who were interested in probing why contemporary U.S. college students do poorly in folk-biological tasks. The authors assessed the temporal dynamics of folk-biological knowledge encoded in common English using a digitized historical dictionary of the English language. The key finding was that cultural conceptual knowledge about trees evolved from the 16th to 19th century, but sharply declined during the 20th century. This analysis was limited to dictionary entries, but researchers can now explore a wider range of texts with the time-stamped ngram corpora from Google (Michel et al., 2011), making historical comparisons easier to implement and more precise in detecting year-to-year changes. The ngram database is featured in Greenfield's (2013) study of cultural change in American and British values during the last two centuries, where she found a stable decline of words related to duties, obligations and belonging, accompanied by increases in words related to individualism, choice, and materialistic values.

Applying automated text analysis to study historical trends in causal cognitive processes may be more challenging than studying changes in knowledge content or social values, which seem more amenable to direct analysis via word frequencies. Still, some applications might be straightforward. For example, LIWC offers a causality-focused dictionary that could be used to measure cultural shifts in the frequency of causal language. Cognitive psychologists interested in historical trends can also develop specialized dictionaries guided by particular cognitive theories. Such an approach was used by Dehghani et al. (2013) to compare cultural epistemologies in Native American and majority-culture American children's books. This strategy is particularly useful when the objective is to distinguish between multiple cultural views on causality, rather than studying causal thinking as a unitary construct.

## Conclusion

The field of cultural cognition traces its foundations to scholars who treated culture and history as complementary constructs (Vygotsky, 1978). Yet most subsequent empirical work in the field has been focused on cross-cultural comparisons alone, rather than diachronic analysis within cultures. Accounting for historical trends in cultural cognition will not only demand new methodological developments, but will also press us to apply a more dynamic concept of culture (see Brumann, 1999; ojalehto and Medin, 2015). Theoretically, researchers are challenged to consider whether “culture” is as much a temporally as it is a spatially (and politically, economically, linguistically) bounded construct. Treating culture as a dynamic system of social, ecological, economic, institutional and psychological factors will complicate our task, but it will also bring new insights and deeper understanding of the complex interaction between culture and cognition.

### Conflict of interest statement

The authors declare that the research was conducted in the absence of any commercial or financial relationships that could be construed as a potential conflict of interest.

## Footnotes

^1^For some notable exceptions see Barrett et al. (2005), Beller et al. (2009), Abarbanell and Hauser (2010), and Bender and Beller (2011).
